# Special Issue on Advanced Optical Technologies for Communications, Perception, and Chips

**DOI:** 10.3390/s25175278

**Published:** 2025-08-25

**Authors:** Tianxu Xu, Kaiheng Zou, Cong Liu, Yang Yue

**Affiliations:** 1 School of Electrical and Information Engineering, Zhengzhou University, Zhengzhou 450001, China; 2 Department of Electrical Engineering, University of Southern California, Los Angeles, CA 90089, USA; kai-hengz@usc.edu (K.Z.); liucong@usc.edu (C.L.); 3 School of Information and Communications Engineering, Xi’an Jiaotong University, Xi’an 710049, China; yueyang@xjtu.edu.cn

## 1. Introduction

With the iterative upgrade and popular application of new information technologies such as 5G, cloud computing, big data, and artificial intelligence (AI), the global data traffic and the demand for computing power has ushered in explosive growth. Traditional data transmission speed, information capacity, and chip computing performance can no longer meet the processing needs of big data [1]. Recent progress in optical technologies has highlighted a feasible development route for these contradictions. In particular, optical communication, optical perception, and optical chips are considered to be the most promising research directions to explore. At present, optical communication focuses on core technical issues, including larger bandwidth, lower latency, and smaller packet loss rate, to ensure that high-quality networks meet and support emerging applications (e.g., 4K/8K broadcasting, VR/AR, and free-view video). Optical perception technology has the advantages of convenient acquisition, low cost, and a large amount of information. With the continuous innovation of basic theory and analysis technology, researchers are devoted to developing perception systems, constructing multi-dimensional image knowledge systems, and promoting the development of image theory and application to a deeper level [2,3]. As a design that closely matches the optical computing architecture and the AI algorithm, optical chips offer the advantages of high-speed parallelism and low power consumption and can solve many problems in the application fields such as long processing time and high power consumption [4]. The above-mentioned research covers the core technologies in key fields to provide high speed, wide bandwidth, large volume, and low power consumption.

This Special Issue focuses on the state-of-the-art advancements in optical technologies for communication, perception, and chips, with a specific focus on digital, electrical, and optical signal processing theories, AI, integrated chips, devices, and subsystems/systems. The included papers successfully achieve these goals by contributing cutting-edge design, analysis, computation, and experiments with remarkable results. Two review papers, one communication, and nine research articles comprise the Special Issue, and these curated contributions highlight remarkable innovations while revealing critical knowledge gaps and future research imperatives. Throughout this editorial, numbers in parentheses (e.g., (1), (2), etc.) refer to the contributions in this collection, details of which can be found in the “List of Contributions” section. For clarity, we have grouped the works by topic and summarized the latest results in the field. This editorial is only a brief overview of the outstanding works included, and we encourage readers to explore this Special Issue in full.

## 2. Optical Communication: Speed, Security, and Novel Architectures

In optical communications, the pursuit of ultra-low latency and ultra-high bandwidth continues to drive innovation in fiber and free-space systems. Cao et al. designed a graded-index ring-core fiber (contribution 1), which includes a GeO_2_-doped silica ring core and a SiO_2_ cladding, to achieve near-vacuum optical propagation. Ullah et al. proposed a novel central office (CO) for the optical metropolitan access network (OMAN) with an affordable and unique switching system (contribution 2), thereby facilitating a 1.2 Tbps downlink-capable metropolitan access network. Currently, broader breakthroughs include ultrafast optical time-domain transformation (UO-TDT) techniques achieving MHz-rate imaging through spectral-to-temporal mapping, crucial for real-time monitoring in 5G/6G networks [5]. In pursuit of high-speed optical communication, Zhu et al. adopted a new flexible organic photodetector (OPD) and demonstrated a high data transmission rate of 80 MHz with a bit error rate of 3.5 × 10^−4^, meaning it has great potential in next-generation high-speed flexible optical communication systems [6].

With security being critical to a system, quantum augmentation solutions are moving from theory to practice: Quantum key distribution (QKD) networks have now implemented quantum-secured optical layer encryption (contribution 3). To enhance the confidentiality of visible-light communication (VLC) systems, Shi et al. constructed a distributed LED VLC system with multiple users and an eavesdropper, proposing a tabu search-based LED selection algorithm using perfect channel state information (CSI) to avoid local optima (contribution 4). In addition, a loss-tolerant quasi-quantum secure direct communication (QSDC) protocol was demonstrated by using single photons that simultaneously transmitted information and exchanged keys, achieving a world-record 2.38 kbps secure rate over 104.8 km fiber [7].

## 3. Optical Perception: Sensing, Intelligence, and Applications

In optical perception, computational imaging and AI-driven sensing are overcoming traditional limitations. This Special Issue highlights the first ultrafast time-stretch optical coherence tomography (TS-OCT) system to employ reservoir computing (RC) for direct temporal signal analysis—eliminating the need for Fourier transform techniques. By leveraging the temporal characteristics of interference signals while ignoring frequency chirp, this approach offers an efficient solution to nonlinear wavelength sweeping challenges (contribution 5). Related studies also reflect that computational imaging and sensing are redefining resolution and functional analysis. For example: tip-based ultrafast nano-spectroscopy enables higher levels of spatial resolution with femtosecond temporal precision [8]. Chen et al. proposed a partially coherent optical neural network (PCONN) transmission model using mutual intensity modulation, enabling natural-light-based computational reasoning via simple filtering to achieve end-to-end optical processing from signal acquisition to inference [9]. Li et al. prototyped and integrated an all-optical mechanical sensor that mimics mechano-sensitive hair-like sensilla (MSHS) based on thin-walled glass microbubbles as flexible whispering gallery mode resonators. This mechanical sensor can be used as a real-time, directional mechanical sensing whisker in a quadruped cat-like robot, showing its potential for innovative mechanical transduction, artificial perception, and robotic applications [10]. Meanwhile, spatiotemporal OCT (STOC-T) permits hemo-dynamic monitoring at 112 volumetric frames/sec directly from structural data, by-passing complex flow-processing algorithms [11]. For depth information application, Wang et al. proposed a new non-contact mask recognition method based on time-of-flight (ToF) camera, and can further classify mask types, such as medical masks and N95 masks (contribution 6). Shan et al. developed a hemispherical photoelectric memristor array based on Ag-TiO_2_ nanoclusters/sodium alginate film, which realized depth perception and motion detection based on binocular parallax [12]. To further improve the accuracy of feature extraction, the processing of optical information tends to explore the complementarity of cross-modal features. Through deep learning algorithms, it enhances the robustness of sensing systems to achieve adaptive fusion and intelligent processing of multi-dimensional information [13,14]. In addition, an optical microfiber array skin (OMAS) was reported to mimic the interlaced structure of tactile vesicles for tactile visualization and object reconstruction perception, which was characterized by high sensitivity (−0.83 N/V) and fast response time (38 ms) [15]. These advances mark a shift to computational perception, where AI extracts potential information from optical signals.

## 4. Optical Chips and Computing: Devices and Integration

In the photonic device, Yang et al. compared the effect of output waveguide con-figurations on the performance of array waveguide gratings (AWGs), and found the AWG with an output waveguide converging on the grating circle had larger crosstalk and lower nonuniformity (contribution 7). Based on the above research, a 1 × 8 arc-shaped AWG on a SOI platform with a central operation wavelength around 1550 nm was designed and fabricated. Experimental results indicated that the fabricated AWGs outperform in terms of insertion loss and nonuniformity. For fiber Bragg gratings, Cięszczyk et al. reduced the effect of noise on the results by cumulative pretreatment. This method can be used directly or in combination with other algorithms and is much simpler than the spectrum downgrade method (contribution 8). Krause et al. proposed a pioneering optimal double titanium nitride heater model for thermo-optic phase shifters at 1550 nm using standard silicon-on-insulator technology (contribution 9), which reduces system energy cost by achieving a π-phase shift with only 19.1 mW power, low thermal crosstalk (0.404), and minimal optical losses over 1 mm, addressing MZM imbalance issues for data centers and long-range optical communication transmitters. In addition, Liu et al. designed a graded exponential ring fiber featuring a GeO_2_-doped silica ring core and SiO_2_ coating (contribution 10). The structure suppresses spin–orbit coupling effects, thereby reducing power transfer between modes and enhancing orbital angular momentum (OAM) mode purity. Specifically, the purity of the OAM_1,1_ mode increases from 86.48% to 94.43%.

In the field of optical chips, Li et al. proposed and demonstrated an on-chip directional multiplexing strategy for high-capacity 3D multi-planar projection in free space [16]. By exploring and exploiting the directional dependence of guided wave driven detour phase manipulation, they achieved 16-channel on-chip holography, where different images are encoded independently on different z-planes in four illumination directions. Lin et al. experimentally demonstrated an on-chip device capable of generating optical skyrmions with tailored topological invariants [17], which is a breakthrough in chip technology and brings new light to optics and materials science. Gagino et al. demonstrated an integrated optical phased array (OPA) based on an InP photonic integration platform, embedding built-in optical amplifiers and phase modulators to provide beamforming, gain, and beam steering functions in the 1465–1590 nm wavelength range [18]. It has the highest on-chip gain and output power level among active OPAs (i.e., OPAs with amplification functions). These research works show that optical chips are evolving from a single transmission medium to an integrated platform for intelligent computing and communications. The current research focus has shifted to room-temperature operation mechanisms, high-integration systems, and high-dimensional quantum state regulation, breaking through the limitations of traditional low-temperature environments and free-space optics. It promotes the integrated design of quantum light sources, detectors, and chips, laying a foundation for the scalable application of quantum communication and computing [19,20]. In addition to photonic devices and integration, research continues on developing electro-optical nonlinear materials to achieve low-power, high-speed dynamic optical regulation, and the application areas include ultrafast lasers, topological photonic devices, and integrated optical computing [21,22].

Moreover, Bravo et al. first presented a comprehensive state-of-the-art study on hybrid networks based on radio frequency (RF) and VLC under ideal conditions (contribution 11). The characteristics and implementation of these hybrid networks in outdoor scenarios with adverse conditions are then analyzed. Finally, the main challenges and future directions of these hybrid networks are summarized. Furthermore, Isiaka A. et al. provided a comprehensive overview of the enabling technologies, challenges, trends, and future prospects of FSO communications in the context of 5G and beyond (contribution 12). The atmospheric turbulence (AT), weather-induced signal attenuation, and alignment issues faced by current FSO systems are analyzed, and key enabling technologies such as adaptive optics, modulation schemes, and error correction codes are explored. In addition, the advantages of integrating FSO with emerging technologies are discussed. Finally, the prospects of FSO communications in the evolving 5G and future network landscape are emphasized, and key challenges and future research directions for fully realizing the potential of FSO in next-generation communication systems are identified.

## 5. Conclusions

With the rapid development of the digital economy, the application of advanced optical technology in the fields of communication, perception, and chips has become increasingly important. In terms of optical communications, the scale of the global optical communications market is constantly expanding and maintaining steady growth, and more high-performance networks and devices will be developed. In the field of optical perception, the rapid development of deep learning and artificial intelligence has promoted the continuous breakthrough and iteration of core perception technologies, and has also accelerated the leap from 2D imaging to 3D perception, making optical perception technology a key common technology for intelligent upgrading in each related industry. Optical chips have broad application prospects in emerging fields such as artificial intelligence, high-performance computing, and quantum computing. They can not only improve data transmission speed and reduce energy consumption and costs, but also promote technological innovation and industrial upgrading in relevant fields.

This Special Issue crystallizes critical advancements across optical communication, perception, and chip technologies—each domain propelled by distinct yet interconnected innovations. The collected works not only address immediate technical bottlenecks but also illuminate pathways toward optics-dominated computing and sensing infrastructures. Future breakthroughs will emerge in the areas of device innovation, computational frameworks, and system-level co-engineering. We envision a new era where optical technologies transcend traditional roles, becoming the backbone of intelligent, secure, and sustainable global infrastructures.
